# Efficacy of the Mental Health App Intellect to Reduce Stress: Randomized Controlled Trial With a 1-Month Follow-up

**DOI:** 10.2196/40723

**Published:** 2022-12-14

**Authors:** Sean Han Yang Toh, Jessalin Hui Yan Tan, Feodora Roxanne Kosasih, Oliver Sündermann

**Affiliations:** 1 Intellect Pte Ltd Singapore Singapore; 2 Department of Psychology National University of Singapore Singapore Singapore

**Keywords:** mobile health, mHealth, randomized controlled trial, RCT, self-guided interventions, cognitive behavioral therapy, CBT, stress coping, stress management, university students, psychological mindedness, coping self-efficacy, mobile phone

## Abstract

**Background:**

Excessive stress is a major global health concern, particularly in young adults. Short skills-focused self-guided interventions (SGIs) on smartphones are a scalable way to improve stress-coping skills at the population level.

**Objective:**

In this randomized controlled trial, we aimed to examine the possible efficacy of a recently developed stress-coping SGI (*Intellect*) in improving psychological distress, relative to an active control group and 2 potential moderators of this predicted relationship (ie, psychological mindedness [PM] and coping self-efficacy [CSE]).

**Methods:**

University students (N=321) were randomly assigned to either an 8-day SGI on stress-coping or an active control group. Self-reported measures were obtained at baseline, after the intervention, and at the 1-month follow-up. The primary outcome was psychological stress (Psychological Stress Measure-9). Secondary outcomes were anxiety (Generalized Anxiety Disorder-7) and depressive symptoms (Patient Health Questionnaire-9). PM and CSE were assessed as potential moderators at baseline.

**Results:**

The final sample (n=264) included 188 (71.2%) female, 66 (25%) male, 7 (2.7%) nonbinary, and 3 (1.1%) others participants with a mean age of 22.5 (SD 5.41) years. The intervention group reported significantly lower perceived stress (partial eta–squared [*ηp*^2^]=0.018; *P*=.03) and anxiety (*ηp*^2^=0.019; *P*=.03) levels after intervention relative to the active control group. The effects on perceived stress levels remained statistically significant at the 1-month follow-up (*ηp*^2^=0.015; *P*=.05). Students with the lowest CSE and highest PM experienced the fastest decline in perceived stress levels (β=6.37, 95% Cl 2.98-9.75). Improvements in anxiety levels were not observed at 1-month follow-up. Similarly, no intervention effects were found for depression levels at postintervention and follow-up periods.

**Conclusions:**

This study provides evidence that the *Intellect* stress-coping SGI is effective in reducing perceived stress and anxiety levels among university students. Mobile health apps are brief, scalable, and can make important contributions to public mental health.

**Trial Registration:**

ClinicalTrials.gov NCT04978896; https://www.clinicaltrials.gov/ct2/show/NCT04978896

## Introduction

### Background

Psychological stress occurs when one’s perceived demands exceed one’s perceived capacity to cope [1,2]. Excessive and prolonged stress can have multiple negative health effects, including heightened risk of obesity [3], impaired working memory [4,5], and cardiac arrest [6]. Adverse stress effects on mental health are also common, including an increased risk of depression and anxiety [7], insomnia [8], and psychosis [9]. Excessive stress is widely recognized as a major health burden [10]. Young adults and late adolescents are at particular risk of experiencing heightened stress levels owing to life transitions from childhood to adulthood and taking on new responsibilities [11,12] or uncertainties relating to career prospects [13]. Equipping young adults with effective stress-coping skills is a viable attempt to lower the public burden of excessive stress [9]. Digital tools such as mobile health (mHealth) apps are likely going to play an increasingly important role in public mental health [14].

Self-guided interventions (SGIs) on smartphones are easily accessible, affordable, flexible, and convenient to use [15]. SGIs can also promote well-being and prevent illness by equipping individuals with the skills to address minor stressors before they evolve into larger problems [16]. Various randomized controlled trials (RCTs) have examined the efficacy of SGIs on mental health outcomes [17], and meta-analyses have confirmed that SGIs can improve symptoms of anxiety and depression across age groups [18,19]. A recent meta-analysis by Linardon et al [20] specifically found that SGIs were efficacious in reducing stress levels. However, the effect sizes for most interventions on stress (Hedges *g*=0.35), anxiety, and depression were rather small (Hedges *g*=0.19-0.21) and inconsistent across studies [18,19]. Although many SGIs are freely available on smartphones, only a few are supported by theoretical and empirical data [21]. In a review of 62 stress management SGIs [22], a quarter of them did not use any evidence-based stress reduction strategies. These results correspond to a separate review of 60 stress-coping SGIs whereby a third of them either failed to provide any empirical strategies or delivered a different strategy than that stated in its description [23]. In both reviews, most of the remaining SGIs focused on momentary breathing and mindfulness exercises, which produced short-term modest effects on stress and anxiety levels. Only a minority of SGIs involved evidence-based interventions such as cognitive behavioral therapy (CBT) [21,23]. Indeed, the meta-analysis by Linardon et al [20] found substantially larger effect sizes for SGIs that involved CBT-based practices compared with waitlist control group. These CBT-based effects on stress, anxiety, and mood were also superior to alternative interventions and could be strengthened when combined with brief professional guidance within the mHealth app. The same authors also concluded that research on the overall efficacy and dissemination of evidence-based SGIs is in its infancy because of the small number of studies, especially those involving younger adults (ie, 5 studies). In addition, the meta-analysis by Linardon et al [20] highlighted methodological problems with RCTs involving stress-coping SGIs, such as high risk of bias and lack of follow-up data.

Furthermore, very little is known about who benefits the most from stress-coping SGIs [24]. Previous authors were unable to analyze any moderators of web- and computer-based stress interventions owing to the “lack and inconsistency of information provided by the [reviewed] studies” [25]. To our knowledge, only Coudray et al [26] evaluated moderators in a sample of 920 college students and found that those with lower perceived present control (ie, aspects of stressors perceived to be controllable in the present) experienced significantly greater reduction in stress after 1 to 3 weeks of web-based stress management interventions compared with those with higher perceived present control. The authors concluded their findings by encouraging similar research on moderators using diverse samples. A potential moderator that may influence the efficacy of CBT-based SGIs on mental health outcomes is coping self-efficacy (CSE). CSE refers to one’s confidence in coping strategies [27], particularly in times of hardship [28,29] and threat [30]. Slightly different from perceived control, which refers to one’s perception of the availability of any effective response, CSE describes one’s confidence in their ability to actually effect that response [31]. Some RCTs have found CSE to be a mediator of the relationship between CBT-based SGIs and improved stress, anxiety, and depression outcomes [32-34], but researchers are less clear about CSE as a moderator. For instance, individuals with higher perceived confidence in coping with stressors were expected to maximize their gains from CBT-based treatments [35], or similar to the findings on perceived present control, it is also possible for individuals who perceive that they need more help with coping have higher motivation to benefit from using a CBT-based SGI, relative to those with higher CSE. Another potential moderator of the relationship between CBT-based SGI and outcomes is psychological mindedness (PM). PM refers to one’s predisposition to be aware of, assess, and reflect on one’s mental states and behaviors cognitively and emotionally [36,37]. Self-reflective practices within CBT were involved in improving depression symptomatology in depressive disorders [38], anxiety disorders [39], and social phobia [40], as it is thought to enhance individuals’ abilities and frequency in self-monitoring and self-evaluating their cognitions, emotions, and behaviors. Despite PM being central to the successful practice of CBT [41], very little is known about the differences in an individual’s capacity for self-reflection and insight that could moderate the efficacy of the modality [36,42]. Wiles et al [43], for example, did not find PM to be a treatment moderator in CBT, although they acknowledged that their small sample size was inadequate for testing interaction effects. Some studies have recommended higher initial levels of self-reflection and insight that may predict faster responses to CBT as potential avenues for future research [36,42]. These authors speculated that metacognitive processes such as higher PM might predispose an individual’s capacity to appreciate and understand the cause-and-effect concepts of CBT-based techniques. As CBT principles are often adopted by evidence-based SGIs, it would be interesting to test how initial levels of PM can influence CBT-based therapy outcomes, as it would allow personalizing CBT-based SGI and optimizing treatment outcomes.

### Objectives

Using a randomized controlled design, we examined the possible efficacy of a recently developed CBT-based stress-coping SGI in the mHealth app (*Intellect*) on a sample of Singaporean university students compared with an active control group. We first hypothesized that participants in the intervention group (stress SGI) would report lower perceived stress than the active control group after the intervention. Although the effects of self-guided CBT interventions for stress coping are typically small, we predicted possible gains for the intervention group to be maintained at 1-month follow-up, in line with related RCTs that also found small effects present at follow-up assessments [44,45].

Second, we predicted that participants in the intervention group would report lower anxiety and depressive symptoms than the active control group after the intervention. Third, we hypothesized that users with higher levels of PM and CSE would experience the greatest reduction in perceived stress levels. As studies have shown that individuals who benefited most from stress interventions typically also experienced greater improvement in anxiety and depressive symptoms [46], we also predicted that this moderation effect by CSE and PM also applies to secondary outcomes.

## Methods

### Study Design

This study was an RCT with two groups: (1) an 8-day stress-coping self-guided program (intervention group) and (2) an 8-day cooperation self-guided program (active control group). A 2 × 3 mixed factorial experimental design was used with *condition* (intervention vs active control) as a between-group factor and *time of assessment* (baseline vs postintervention vs 1-month follow-up) as a within-group factor. The primary dependent variable measured was perceived stress levels. The secondary dependent variables were depression and anxiety levels. The CSE and Psychological Mindedness Scale (PMS) were also used for independent moderation analyses.

### Recruitment and Study Participants

**Figure 1:**
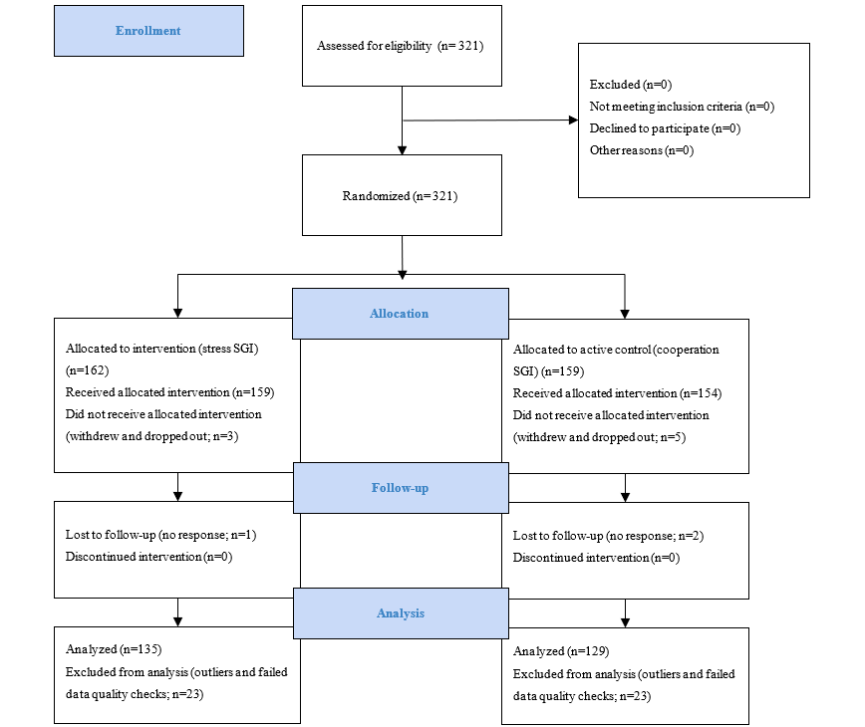
CONSORT (Consolidated Standards of Reporting Trials) flowchart. SGI: self-guided intervention.

A total of 321 participants were recruited through the Psychology Department’s Research Participation Programme and the university’s research recruitment platform. Recruitment posters comprising the study procedures, inclusion criteria, and reimbursement for participation were distributed on the web and among the faculty. Of the 321 participants, 46 (14.3%) participants were excluded owing to withdrawal and failing data quality checks, whereas 3 (0.9%) participants were lost to follow-up (Figure 1). Of the 321 participants, the final sample of 264 (82.2%) participants was predominantly female (188/264, 71.2%), with a mean age of 22.45 (SD 5.41; range 18-59) years. All the participants were undergraduate students who were able to read and understand English. Elevated perceived stress was not a requirement for inclusion. Participants also received either course credits or monetary reimbursement for their participation.

### Power Calculation

Most studies that investigated app-based stress-coping programs found small to moderate effect sizes for the primary outcome measures of stress, mood, and anxiety [47]. Subjecting a small effect size of Cohen *f*=0.1, an α level of .05, and a power of 0.9 to G*Power revealed a minimum sample size of 214. Web-based studies are prone to attrition, and in line with similar studies, we expected 30% to 50% attrition rate [47,48] and considered that 10% of the questionnaire data may be inconsistent or invalid as commonly found in web-based survey studies [49]. Thus, we aimed to recruit 321 participants.

### Procedure

Participants signed up for the study on the university’s recruitment sites via a link that directed them to the web-based survey platform hosted on Qualtrics (SAP America Inc). Upon consenting to the web-based Participants Information Sheet, participants completed the primary outcome measure on perceived stress (Psychological Stress Measure [PSM]; PSM-9), secondary outcome measures on mental health (Patient Health Questionnaire [PHQ]; PHQ-9 and Generalized Anxiety Disorder [GAD]; GAD-7), CSE (Coping Self-Efficacy Scale [CSES]), PMS, and demographic information.

Next, participants were randomly assigned to either the intervention or active control condition using simple randomization procedures through the Qualtrics software. They were not informed about the conditions they were allocated to and the real nature of the study. Instead, they were informed that the study would examine the efficacy of SGIs in promoting well-being. They were then guided to download the mHealth app *Intellect* on their personal smartphones from the Apple App Store (Apple Inc) or Google Play Store (Google LLC). According to their assigned conditions, a number code was provided to them to unlock the app. Participants in the intervention condition participated in the 8-day stress-coping program, whereas those in the active control condition took part in the 8-day cooperation learning program. Both programs involved fulfilling a series of tasks aimed at improving well-being. This included content education and short daily activities, averaging 5 minutes. To promote adherence to the study, standardized daily reminders to complete the program were sent via SMS text messages by the researcher to participants. Participants were only allowed to proceed onto the next page of the app after they had completed the preceding exercises. It was expected that every participant would complete all the self-guided daily activities. All the participants were instructed to refrain from using any other SGIs that affect well-being other than the given SGI throughout the entire duration of the study, lasting from the beginning until the end of the 1-month follow-up. This minimized the potential confounding effects.

Upon completion of the 8-day program through technical verification, participants received a survey link to complete the outcome measures and the App Engagement Scale (AES). A month after the completion of the SGI, participants were provided with a survey link to complete the outcome measures. Reimbursement was given upon completion of the postintervention measures with either 3 course credits or Singapore $10 (US $7.5) and after 1-month follow-up measures with an additional 1 course credit or Singapore $5 (US $3.75). University students intending to major in psychology were required to collect a minimum of 28 course credits over the course of an academic year. After the follow-up assessment, participants in the active control group were also given access to the stress-coping SGI.

### Interventions

#### Stress-Coping Program

This was an 8-day program that provided psychoeducation on the negative effects of stress and effective stress management skills. Table 1 provides an overview of the 6 topics and content covered. Guided by the principles of CBT, the program helped users to identify and change unhelpful thought patterns and behaviors related to stress. Participants engaged in a series of daily exercises involving reflection and mindfulness, and they were guided to identify and type down their stressors, negative thoughts associated with the stressors, and positive affirmations. Participants were also taught breathing exercises and encouraged to practice them (Table 1 presents an overview of the program).

**Table 1:** Overview of stress-coping program.

Topic	Content
Topic 1: How stress affects the body	Be able to identify personal stressors Understand the difference between internal and external stressors
Topic 2: Saying “no” to burnout	Understand the impact of stress on well-being Understand and identify emotional, physiological, and behavioral signs and symptoms of stress
Topic 3: Learning to manage stress	In-depth introduction of stress Understand that stress can be healthy if kept at manageable levels
Topic 4: Self-care of the mind	Understand that internal stressors are self-induced feelings and thoughts Be able to identify negative thoughts that lead to stress Develop skills to challenge negative thoughts Develop skills to engage in positive affirmation
Topic 5: Self-care of the body	Understand that external stressors are events or situations caused by the environment Understand the importance of prioritizing one’s own well-being in stressful situations that cannot be controlled Develop skills in communicating negative feelings in stressful situations Be able to manage stress with the help of deep breathing exercises
Topic 6: Continuing daily self-care	Recap of the entire learning path and key information in each learning session

#### Cooperation Learning Program

The 8-day program on cooperation functioned as the active control group. This program provided psychoeducation through 5 topics for participants to understand and improve collaboration and interpersonal relationships. Short quizzes and feedback-giving exercises were included (Table 2 provides an overview of the program). The duration of the cooperation SGI matched the stress SGI in terms of time and effort required to complete the program.

**Table 2:** Overview of cooperation program.

Topic	Content
Topic 1: What is “cooperation”?	Be able to identify personal preferences to cooperation Understand the differences between cooperation and conformity
Topic 2: Focusing on the bigger picture	Cultivate awareness of collective goals Be able to identify possible strengths in a team member that can propel toward the collective goal Be able to identify possible weaknesses in a team member that can hinder team efforts
Topic 3: Understanding group dynamics	In-depth introduction of group dynamics Be able to identify the correct scenarios that support or threaten group dynamics
Topic 4: Building positive relationships	Understand that healthy relationships, which strengthen group dynamics, are important drivers toward attaining team’s objectives Learn 2 methods that can build empathy and trust to improve group relationships: support and feedback Offering support: develop skills to manage tone, body language, and learn a set of encouraging words Offering support: identify ways to empower team members’ strengths and to cover each other’s weaknesses or blind spots Providing feedback: in-depth introduction of the many ways one can provide constructive feedback to team members
Topic 5: Learning to work together	In-depth recap of the entire learning path and key information in each learning session

### Measures

#### Primary Outcome Measure

PSM-9 [50,51] measures the affective, cognitive, behavioral, and somatic components of psychological stress. This is a 9-item self-report measure using an 8-point scale (ranging from 1=“Not at all” to 8=“Extremely”). Some examples of the items include “I feel calm” and “I feel a great weight on my shoulders.” As the midpoint of this scale is 4=“A bit,” an overall score of ≥36 on this measure would indicate that the overall sample is at elevated stress. Higher total scores reflect more stress symptoms. The PSM-9 has an acceptable internal consistency with Cronbach α ranging from .74 to .78 in this study.

#### Secondary Outcome Measures

##### Patient Health Questionnaire

PHQ-9 [52,53] is a widely used 9-item measure of depression symptoms. The PHQ-9 was included in this study as stress symptoms often include low mood and depression. Items are scored on a 4-point scale (ranging from 0=“Not at all” to 3=“Nearly every day”), with higher scores indicating more depressive symptoms. The total score of the PHQ-9 ranges from 0 to 27, with scores of 5, 15, and 20 indicating the cutoff points for mild, moderate, and severe depression, respectively [53]. The internal consistency of PHQ-9 in this study was very good with Cronbach α ranging from .86 to .88.

##### Generalized Anxiety Disorder

GAD-7 [54] is a 7-item self-report instrument that measures symptoms of generalized anxiety. We included the GAD-7 because general anxiety is associated with heightened stress levels. Similar to the PHQ-9, it uses a 4-point scale (ranging from 0=“Not at all” to 3=“Nearly every day”), with higher scores indicating more severe symptoms. Mild, moderate, and severe anxiety were indicated by scores ranging from ≤9, 10-14, and 15-21, respectively [55]. The internal reliability of the GAD-7 in this study was excellent with Cronbach α ranging from .90 to .91.

##### Coping Self-Efficacy Scale

CSES [28] is a 26-item scale that assesses perceived self-efficacy for coping with threats and challenges. Items include “Keep from feeling sad” and “Resist the impulse to act hastily when under pressure.” An 11-point scale was used (0=“Cannot do at all”; 5=“Moderately certain can do”; 10=“Certain can do”), with higher scores reflecting a stronger belief in one’s coping abilities. In this study, CSES possessed an excellent internal consistency of Cronbach α=.90.

##### Psychological Mindedness Scale

PMS [37] measures an individual’s ability to reflect on psychological processes, emotional processing, and interpersonal relationships. The PMS is a 45-item self-report instrument consisting of items such as “I often find myself thinking about what made me act in a certain way” and “I am sensitive to the changes in my own feelings.” It uses a 4-point scale to score items (1=“Strongly disagree”; 4=“Strongly agree”). Higher total scores indicated higher levels of PM. The PMS produced a good internal consistency of Cronbach α=.84 in this study.

##### App Engagement Scale

The AES [56] assesses the degree of app engagement, which was included to assess whether both groups were equally engaged in the SGIs. This 7-item scale uses a 5-point Likert scale (ranging from 1=“Strongly disagree” to 5=“Strongly agree”). The scale had good internal reliability in this study, with a Cronbach α=.85. App engagement was predictive of positive outcomes on measures of mood and anxiety [57].

### Statistical Analyses

#### Data Screening

Incomplete responses were excluded from the study. Following the multiple hurdles approach by Curran [58], the most likely invalid data were sequentially identified in 2 steps and removed. First, submissions with an overall response time of <800 seconds were flagged, as it indicated that participants sped through the questionnaire and downloading the app, suggesting that they may not have participated properly. The subsequent checks confirmed whether the flagged responses were invalid. Next, data with strings of identical responses (eg, selecting “agree” to all items) for the entire scale of any of the self-report measures were excluded. Subsequently, several attention checks were used in the self-report scales [58]. For example, in the PSM-9, items 1 and 6 were reverse-coded. A contradiction was detected in the participants’ self-report if responses to these 2 items were congruent with the other items on the scale [51]. Similarly, in PMS, responses to items 5 and 23 should be similar to each other but opposite to that of item 35 [37]. Contradictory responses were considered invalid and were subsequently removed. Importantly, removing these responses did not change any outcomes significantly. All statistical analyses were performed using IBM SPSS Statistics (version 25.0, IBM Corp). Data were first visually inspected using scatter plots and histograms to examine the distribution of the data and identify significant outliers. Visual inspection of these data revealed a normal distribution within an acceptable range of skewness and kurtosis (all values between −1 and 1), in accordance with the guidelines of Kline [59]. In addition, 5 outliers (ie, participants who reported data further than 2.5 SDs from the mean) were removed before the analyses [60,61]. Independent 2-tailed *t* tests and chi-square tests were used to examine any baseline differences of all demographic and dependent variables between the intervention and control groups. Should group differences of these variables emerge, they would be controlled for in the statistical comparisons.

#### Main Analyses

Analysis of covariance (ANCOVA) examined changes at postintervention and follow-up periods between the intervention and active control groups. ANCOVA is the recommended analysis for inferential testing of intervention effects [57], as it controls for baseline scores to ensure that group differences are due to intervention effects [62]. The α level was set at *P*<.05. Partial eta–squared (*ηp*^2^) was the effect size reported for ANCOVA, whereas eta-squared (*η*^2^) was the effect size reported for 2-tailed *t* tests and ANOVA. Guidelines by Cohen [63] for eta-squared were used, whereby 0.01 to 0.05 indicates small effect, 0.06 to 0.13 indicates moderate effect, and ≥0.14 indicates large effect.

#### Moderation Analyses

**Figure 2:**
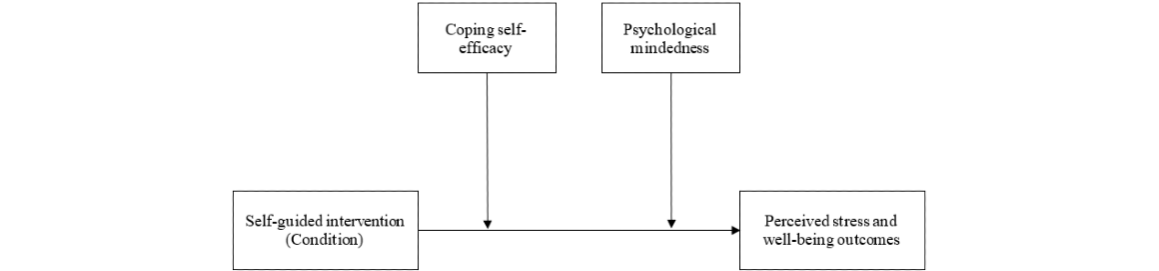
Hypothesized relationships with coping self-efficacy and psychological mindedness as independent moderators of the direct effect of self-guided intervention on mental health outcomes.

Finally, double moderation analyses were conducted using Hayes PROCESS (version 4.0, IBM Corp, macro Model 2; Figure 2) [64]. A total of 6 separate models were conducted. The first 3 models used the primary (ie, PSM-9) and secondary outcome measures (ie, GAD-7 and PHQ-9) as dependent variables after the intervention. The remaining 3 models used the same outcome measures as dependent variables at follow-up. Each model ran a multiple linear regression double moderation analysis to examine the moderating effects of CSE and PM on the relationship between condition (intervention and control) and PSM-9 or PHQ-9 or GAD-7 at postintervention or follow-up period. All analyses were conducted with the respective outcome measures before the intervention as covariates. Each moderator level was determined by the SD value of 1 from the mean. CIs were set at 95%, with 5000 bootstrap iterations to assume normality [65].

### Ethics Approval

Ethics approval to conduct the study was obtained from the Institutional Review Board of the National University of Singapore (NUS-IRB-2021-85). The study was preregistered with ClinicalTrials.gov (NCT04978896). All the participants provided electronic consent before participating in the study. Data collection took place in an entirely web-based setting, and all user data were deidentified before any analyses.

## Results

### Participants

The descriptive statistics for both groups are presented in Table 3. Independent 2-tailed *t* tests and chi-square tests did not find any significant differences in age, sex, and baseline scores for PSM-9, PHQ-9, GAD-7, CSES, and PMS (all *P*>.05) between groups. Participants in both conditions did not show any significant differences in their degree of engagement with their SGIs (*P*>.05) as measured with the AES. The mean scores for the whole sample’s characteristics were as follows (PSM-9: mean 37.78, SD 9.58; PHQ-9: mean 7.07, SD 5.27; GAD-7: mean 6.60, SD 4.57; CSES: mean 142.9, SD 42.2; and PMS: mean 126.2, SD 10.5), indicating that the average student was mildly distressed, depressed, and anxious.

**Table 3:** Descriptive statistics for demographics and outcome variables by condition (n=264).

Variables		Intervention condition (n=135)	Active control condition (n=129)	*P* value	
Age (years), mean (SD)		22.88 (6.26)	22.00 (4.34)	.19	
**Sex, n (%)**					.50
	Female	94 (70.1)	94 (72.9)		
	Male	38 (27.7)	28 (21.7)		
	Nonbinary	3 (2.2)	4 (3.1)		
	Others	0 (0)	3 (2.3)		
PSM-9^a^, mean (SD)		38.5 (9.72)	37.0 (9.40)	.19	
GAD-7^b^, mean (SD)		6.81 (4.62)	6.37 (4.52)	.43	
PHQ-9^c^, mean (SD)		7.35 (5.11)	6.78 (5.44)	.38	
CSES^d^, mean (SD)		139.5 (45.1)	146.5 (38.9)	.18	
PMS^e^, mean (SD)		125.6 (10.4)	126.9 (10.7)	.31	
AES^f^, mean (SD)		27.5 (3.30)	27.2 (3.68)	.56	

^a^PSM-9: Psychological Stress Measure-9.

^b^GAD-7: Generalized Anxiety Disorder-7.

^c^PHQ-9: Patient Health Questionnaire-9.

^d^CSES: Coping Self-Efficacy Scale.

^e^PMS: Psychological Mindedness Scale.

^f^AES: App Engagement Scale (collected after intervention).

### Outcome Evaluations

The mean values of the outcome measures are listed in Table 4. The intervention group reported significantly lower perceived stress levels at postintervention and follow-up periods compared with the active control group. The small effect sizes were between 0.015 and 0.019. This indicated that the stress-coping SGI was moderately more effective than the cooperation SGI (control) in reducing perceived stress over time. The intervention group also reported significantly lower anxiety levels than the active control group at postintervention period but not at follow-up. No significant differences were observed between the intervention and active control groups for depressive symptoms at the 2 time points.

For the ANCOVA results (controlling for all baseline measurements) at postintervention period, the time × group interaction exhibited significant differences for PSM-9 (*F*_1,261_=7.16; *P*=.008) and GAD-7 (*F*_1,261_=5.86; *P*=.02) but not for PHQ-9 (*F*_1,261_=1.65; *P*=.20). Only the interaction effect for PSM-9 (*F*_1,261_=6.08; *P*=.01) remained significant at follow-up (GAD-7: *F*_1,261_=.796; *P*=.37; and PHQ-9: *F*_1,261_=3.24; *P*=.07). Therefore, the main effects on psychological stress scores indicated a significant intervention effect over time.

**Table 4:** Means (SDs), univariate F values, and effect sizes (ESs) for outcome variables at postintervention and 1-month follow-up.

Variable	Baseline		Postintervention						Follow-up				
	Intervention, mean (SD)	Control, mean (SD)	Intervention, mean (SD)	Control, mean (SD)	*F* test *(df)*	*P* value	ES^a^	Intervention, mean (SD)		Control, mean (SD)	*F* test *(df)*	*P* value	ES
PSM-9^b^	38.5 (9.72)	37.0 (9.40)	32.5 (9.41)	33.7 (9.16)	4.82 (1,261)	*.03* ^c^	.018	33.7 (8.89)		34.8 (9.70)	3.89 (1,261)	*.05*	.015
GAD-7^d^	6.81 (4.62)	6.37 (4.52)	5.47 (3.94)	5.95 (4.47)	4.99 (1,261)	*.03*	.019	5.92 (4.33)		5.90 (4.22)	0.094 (1,261)	.76	.000
PHQ-9^e^	7.35 (5.11)	6.78 (5.44)	5.76 (4.29)	5.78 (4.36)	0.613 (1,261)	.43	.002	5.73 (4.23)		6.06 (4.54)	1.80 (1,261)	.18	.007

^a^ESs of 0.01=small, 0.06=moderate, 0.14=large [63].

^b^PSM-9: Psychological Stress Measure-9.

^c^Italicized values indicate significant *P* values at .05.

^d^GAD-7: Generalized Anxiety Disorder-7.

^e^PHQ-9: Patient Health Questionnaire-9.

**Figure 3:**
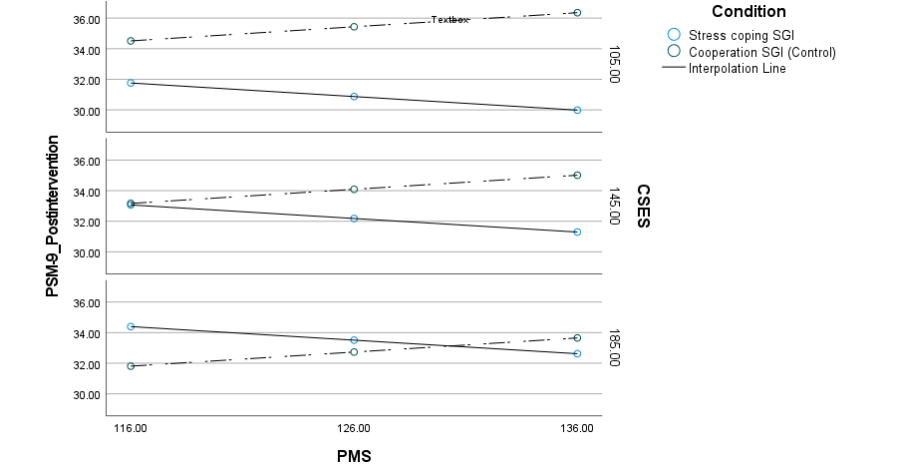
Interactions between SGIs and moderators on psychological stress at postintervention. CSES: Coping Self-Efficacy Scale; PMS: Psychological Mindedness Scale; PSM-9: Psychological Stress Measure; SGI: self-guided intervention. Mean of PMS is 126 (SD 10). Mean of CSES is 145 (SD 40).

At the postintervention period, the results revealed a significant effect in the overall moderation model with PSM-9 as the dependent variable and CSES and PMS as moderators (*R*²=0.39; *F*_9,253_=18.3; *P*<.001). Both interaction terms were significant at postintervention period (CSES × condition: b=−0.0655, SE=0.0231; t_263_=−2.83; *P*=.005; 95% Cl −0.111 to −0.020 and PMS × condition: b=0.181, SE=0.0917; t_263_=1.97; *P*=.05; 95% Cl 0.0002-0.362), indicating that these specific conditions must be met for the intervention condition to predict lower psychological stress [66]. The stress-coping SGI significantly predicted lower scores on the PSM-9 at postintervention period in participants who (1) experienced lower CSE and (2) had moderate to high baseline PM (low CSE and moderate PM: b=4.56, SE=1.30; t_263_=3.52; *P*<.001; 95% Cl 2.01-7.11 and low CSE and high PM: b=6.37, SE=1.72; t_263_=3.71; *P*<.001; 95% Cl 2.98-9.75). Similarly, the intervention condition significantly predicted lower psychological stress at postintervention period for participants with (1) moderate CSE and (2) moderate to high baseline PM (moderate CSE and moderate PM: b=1.91, SE=0.920; t_263_=2.07; *P*=.04; 95% Cl 0.0953-3.72 and moderate CSE and high PM: b=3.72, SE=1.27; t_263_=2.92; *P*=.004; 95% Cl 1.21-6.22). The results are presented in Table 5. Finally, regardless of the level of PMS, participants who began the stress-coping SGI with high SCE did not experience significantly lower psychological stress at postintervention period. These moderation effects were not observed at follow-up, even as the stress-coping SGI group reported significantly lower perceived stress levels at postintervention period (CSE × condition: b=−0.0415, SE=0.0241; t_263_=−1.72; *P*=.09; 95% Cl −0.0891 to 0.0060 and PMS × condition: b=−0.0071, SE=0.0958; t_263_=−.0740; *P*=.94; 95% Cl −0.196 to 0.182). A visualization of these interactions is shown in Figure 3.

**Table 5:** Intervention condition predicts lower psychological stress at each level of the moderators.

Coping self-efficacy	Psychological mindedness	*t* test *(df)*	β (SE; 95% CI)	*P* value
Low (–1 SD)	Low (–1 SD)	1.91 (262)	2.75 (1.44; –0.090 to 5.60)	.06
Low (–1 SD)	Moderate	3.52 (262)	4.56 (1.30; 2.01 to 7.11)	*<.001* ^a^
Low (–1 SD)	High (+1 SD)	3.71 (262)	6.37 (1.72; 2.98 to 9.75)	*<.001*
Moderate	Low (–1 SD)	0.075 (262)	.099 (1.32; –2.51 to 2.71)	.94
Moderate	Moderate	2.07 (262)	1.91 (0.920; 0.095 to 3.72)	*.04*
Moderate	High (+1 SD)	2.92 (262)	3.72 (1.27; 1.21 to 6.22)	*.004*
High (+1 SD)	Low (–1 SD)	–1.44 (262)	–2.59 (1.79; –6.11 to 0.940)	.15
High (+1 SD)	Moderate	–0.581 (262)	–0.778 (1.34; –3.41 to 1.86)	.56
High (+1 SD)	High (+1 SD)	0.719 (262)	1.03 (1.43; –1.79 to 3.86)	.47

^a^Italicized values indicate significance.

Moderation analyses for the secondary measures were not significant. For postintervention and follow-up periods, the interaction terms for both CSE and PM were not significant for both anxiety and depressive outcomes. This means that even as participants in the stress-coping intervention group experienced significantly lower anxiety levels than active control participants at postintervention period, no subgroup of individuals benefited more than the others. Similarly, as there were no significant main effects on anxiety and depressive symptoms at follow-up; there was no moderation effect.

## Discussion

### Principal Findings

#### Overview

This study aimed to evaluate the possible efficacy of an 8-day stress-coping SGI compared with an active control in improving perceived stress and well-being levels in a sample of Asian university students. We also examined PM and CSE as factors that may identify people for whom the SGI worked best. Our hypotheses were largely supported, with the intervention group showing significantly greater improvement in perceived stress levels at postintervention and 1-month follow-up periods. In particular, students with low to medium CSE or medium to high PM benefited from significantly lower perceived stress levels after the intervention than other participants. These moderation effects were no longer observed at follow-up, indicating that these subgroups reaped the benefits of the stress-coping SGI faster than others. There were no additional benefits for participants who began the stress SGI with high CSE. In addition, the intervention group reported significantly lower anxiety levels than the active control group after the intervention without any moderation. The SGIs were also perceived to be satisfactory by both groups of participants. Participants were equally likely to continue using the *Intellect* SGI.

#### Psychological Stress

Our results extend the previous dearth of research that a CBT-based stress-coping intervention delivered on a mobile-based platform can be effective in reducing psychological stress in Asian university students. Previous studies that have compared CBT-based mobile interventions with waitlist controls have shown similar results. Self-guided CBT programs such as Calm [67], SMART-OP [68], and BioBase [69] previously helped decrease perceived stress in Western college students using an RCT design. Calm, consumer-based mindfulness meditation app that incorporates various CBT techniques, enhanced concentration and present-moment awareness in students using daily mindfulness meditations. This heightened awareness and focus, integrated with an 8-week cognitive training course, and facilitated the development of balanced thinking in the face of stressors [67]. Likewise, student users of the 6-week SMART-OP intervention had access to a variety of cognitive behavioral content, including stress management psychoeducation and cognitive restructuring exercises. These were further complemented with relaxation skills (ie, focused breathing, guided muscle relaxation, and biofeedback challenge) [68]. BioBase delivered a 4-week course with elements of CBT and self-compassion. The course focused on recognizing stressors and increasing one’s perception of control to address them [69]. Comparably, *Intellect*’s stress intervention comprises very similar CBT-based content. In addition, the stress-coping SGI within *Intellect* demonstrated the efficacy of such content in reducing perceived stress levels despite reduced exposure (ie, 5 minutes per day for 8 days). Our small effect sizes were also consistent with the effect sizes relative to active controls (*g*=0.09) presented in the meta-analyses of Linardon et al [20]. To address the lack of follow-up assessments in mental health mobile interventions [20,25], our study found that reduced perceived stress levels were sustained at the 1-month follow-up. This was in line with the few stress SGI studies that also conducted follow-up assessments for up to 6 weeks [69,70].

#### Anxiety and Depression

Our second hypothesis was partially supported. The stress-coping SGI was effective in reducing anxiety symptomatology among college students only at postintervention. Similarly, a multitude of stress-coping mobile-based interventions have shown reductions in self-reported anxiety [44,71-73] with similar effect sizes (Hedges *g*=0.07-0.29). However, the length of these interventions was longer, ranging minimally from 2 to 11 weeks [20]. These smartphone interventions used either mindfulness-based practices only [71-73] or both mindfulness and CBT [44]. Regardless of their theoretical orientations, these interventions used relaxation techniques, such as deep breathing and mindful awareness, that are also present in the *Intellect* stress-coping SGI. These methods previously provided short-term relief from anxiety symptoms [74,75]. Despite its brief content, the *Intellect* stress-coping SGI reduced anxiety among university students, possibly because of the use of similar relaxation techniques. In contrast, the stress-coping SGI did not reduce the depressive symptoms. This finding contradicted the few CBT-based smartphone interventions reviewed by Linardon et al [20,76-78]. There are several possible reasons for the null findings. First, in comparison with our stress-coping SGI, the course content within these CBT-based interventions ranged from 2 weeks [77] to 2 months [78]. Hence, it is plausible that their content was more exhaustive and also more specifically tailored to address the etiology and maintenance factors of depression [78]. The stress-coping SGI was specifically designed to provide relief from perceived stress symptoms and did not incorporate more diverse content addressing depressive symptoms. Consequently, it is plausible that its efficacy against depressive symptoms would be limited. Second, the efficacies of these interventions were demonstrated against waitlist control [77] or across multiple time points and without a control group [76]. Research has shown that treatment effects of CBT interventions on depressive symptoms were significantly more difficult to detect when there is an active control group [79]. It is conceivable that the use of an active control may have obscured the intervention effect in our study, given that participants in both the intervention group and active control group were significantly less depressed after the intervention (intervention: mean 5.74, SD 4.28; t_134_=−4.85; *P*<.001; and active control: mean 5.78, SD 4.47; t_128_=−3.07; *P*<.01) relative to baseline (intervention: mean 7.35, SD 5.11; and active control: mean 6.78, SD 5.44). Considering that poor social dynamics was a potential risk factor for depression in college students [80], it is plausible that the active control group improved group dynamics, which may have lowered depression levels. This has been observed in previous studies [81]. However, this conclusion cannot be drawn, as we did not evaluate the efficacy of the cooperation SGI against a waitlist control group. Future studies may shed further light on this hypothesis using a 3-arm RCT with a waitlist control group, a neutral active control group (eg, attention control), and the stress-coping SGI. Interestingly, a few similar interventions have also detected anxiety effects but not depression [82] or depression effects but no anxiety effects [47]. It remains possible that certain stress-focused strategies (ie, relaxation techniques) were more effective in reducing anxiety than depressive symptoms. There were no significant group differences in anxiety or depressive symptoms at the 1-month follow-up. Sharing similar characteristics as our sample, previous RCTs evaluating the efficacy of SGIs with nonclinical participants experiencing mild baseline symptoms generally found small to no effects of app use after intervention [33,83-85]. Bakker and Rickard [33] postulated that these participants may have experienced lower motivation to seriously engage with the app, thus reducing their chances of maintaining psychological benefits.

#### Moderators

Our third hypothesis was partially supported. CSE and PM emerged as significant moderators, such that students with the lowest CSE and highest PM faced the greatest reduction in perceived stress levels within the intervention condition at postintervention. There were no benefits for participants with low PM and high CSE. In addition, this moderation effect was no longer observed at follow-up. The finding that perceived stress levels improved relatively quicker within this group of university students was not entirely surprising. Individuals with a greater propensity for self-reflection and insight were more likely to be aware and thus found it easier to look at their stressors from a distance [36]. Coupled with a deeper motivation to explore the cause of their stressors, these facilitated the use of self-guided CBT strategies to resolve them (eg, reappraisal) [86-88]. McCallum et al [89] also showed that higher levels of PM in psychiatric outpatients were associated with more favorable outcomes in short-term interpretive and supportive therapies. In line with the increasing interest in the nonclinical use of CBT-based self-guided strategies, we found that university students with higher PM reaped faster gains with a CBT-based stress-coping intervention. The finding that individuals with lower CSE also experienced faster gains may be counterintuitive at first. In an RCT that compared the efficacies of 3 different CBT-based SGIs, Bakker et al [47] found that CSE was significantly increased in only 2 of them (ie, MoodKit and MoodMission). Further investigation, however, showed that participants had much lower baseline CSE levels in these 2 samples (152.00 and 154.46, respectively, compared with 169.75 in MoodPrism; [56]). Bakker et al [47] did not find any significant group differences in baseline CSE levels. Beyond CBT-based SGIs, other forms of SGIs have also shown potential ceiling effects for CSE, such that a lack of improvement resulted in no mediation of better stress outcomes [90,91]. In comparison to those with high CSE, individuals with lower perceived confidence to destress successfully would naturally be more motivated and invested in seeking additional help [92]. The belief that one could still learn how to cope with stressors of emerging adulthood was also previously suggested to enhance the effectiveness of stress management interventions [93]. Our results suggest that this may be the case. Future studies may further evaluate CSE and PM as factors to optimize treatment delivery for a nonclinical college student population. The finding that CSE and PM did not moderate the efficacy of the stress-coping SGI on secondary outcomes meant that our final hypothesis was not supported. These results were mostly unsurprising, as there was no main effect of the intervention condition on depression at postintervention and follow-up periods and anxiety at follow-up. Other studies have shown that different forms of coping could be distinctly tailored to different mental health outcomes [94]. For example, lower perceived self-efficacy to problem-solving (problem-focused CSE) has been shown to incline one’s need to find ways to destress [92]. Instead, lower perceived self-efficacy to regulate one’s own emotions to the problem (emotion-focused CSE) may then incline one’s own need to seek emotional help and reduce anxiety. A probable reason that perceived stress levels were moderated, but not anxiety and depression, is likely due to the heavy emphasis on problem-solving internal and external stressors throughout the stress-coping SGI. Individuals with a greater capacity for self-reflection may have mostly reviewed their thought processes and behaviors with regard to the sources of their stressors and adapted with the aim of resolving these root causes. This is similar to those who have lower CSE, as they may channel most of their energy toward equipping new skills to manage stress. As the stress-coping SGI in *Intellect* lacks sufficient emphasis on building emotional resources, we encourage researchers to further evaluate the role of these moderators using alternative CBT-based SGIs.

### Strengths and Limitations

The randomized controlled design allowed for causal conclusions [95]. The active waitlist control group also controlled for the effects of attention and other nonspecific factors from the intervention effects, thus strengthening the validity of the results [96]. Finally, significant effects on perceived stress levels at the 1-month follow-up period showed that the stress-coping SGI successfully improved university students’ well-being over a short period. These directly addressed the lack of follow-up measurements as stated in the most recent meta-analyses of mental health SGIs [20,25].

This study had several limitations. First, the actual amount of effort put in to complete the SGI was not controlled for. Data on the duration that participants spent thinking about or practicing the skills learned from the SGI beyond the intervention were not collected. It is plausible that the positive effects could also be explained by other variables that were not assessed during the intervention, such as better campus life or a less stressful curriculum. Second, the actual duration spent on the *Intellect* app was not collected or controlled for. This is a potentially serious concern, as low adherence and inconsistent engagement with SGIs were previously found to limit their positive effects [97]. However, efforts were made to ensure that the participants completed the app activities properly. Initially, the participants were instructed to complete every daily activity present in their respective SGIs during the intervention period. *Intellect* SGIs also did not allow the participants to proceed to the next page until they completed the preceding activities. We encouraged maximum adherence through daily text reminders. Importantly, every final participant was technically verified as having completed the activities in their SGI. We acknowledged that it is possible that the participants in the active control group completed the activities in less time and with less care than those in the intervention group, which would threaten the internal validity of our findings. However, both groups did not differ on the subjective AES, which gives some confidence that groups did not differ in their motivation to engage with the SGIs and the time they spent on them [98]. Nonetheless, future studies should control for the time spent on the SGI and evaluate its relationship with SGI outcomes. Third, subjective self-reports are prone to retrospective recall biases [99]. This limits the accuracy of our findings on perceived stress, anxiety, and depressive symptoms given that they vary from time to time. Therefore, our results should be interpreted with caution. Given that the PSM-9 asked for self-reported psychological stress over the last 4 to 5 days, we are less able to infer whether the stress-coping SGI could ecologically reduce perceived stress among university students without the implementation of momentary assessments. To further strengthen the reliability of these findings, future studies can use daily diary methods to evaluate the real-time efficacy of mobile interventions on university students’ well-being [100]. In place of self-reports, recent studies have recommended behavioral or physiological measures to objectively assess stress symptoms [101]. This method would also reduce the risk of recall bias and enhance the validity of our findings. Fourth, the study design only compared the intervention group with an active control group. The absence of a waitlist control condition prevented the exclusion of the possibility of third variables contributing to the findings, which can be addressed in future studies by including a waitlist control alongside an active control. Fifth, the absence of long-term follow-up assessments (eg, 3 or 6 months) makes it difficult to deduce whether improvements can be sustained over time. Therefore, our study could not evaluate the possible long-term benefits on symptoms of chronic stress. Finally, this study has limited external validity for the student population at large, as our sample contained mainly female students from Singapore with slightly elevated stress, anxiety, and depression levels. Therefore, our positive findings on perceived stress and anxiety symptoms may be generalized to the Western student population or clinical samples. Future researchers may consider replicating our findings with a more diverse college sample while administering even longer-term follow-up assessments.

### Conclusions

In conclusion, this RCT found evidence for an 8-day stress-coping SGI in improving perceived stress and anxiety levels among Asian university students. The effects on perceived stress levels were sustained at 1-month follow-up, but not for anxiety, thus giving some confidence that a brief, time-limited, and CBT-based SGI can maintain its gains on perceived stress. However, depressive symptoms did not decrease. Students with lower CSE and higher PM experienced reduced stress faster than other students in the CBT-based SGI intervention group. The identification of these moderators can optimize the outcome and treatment delivery of such stress-coping mobile apps. Our findings are useful, given the potential for scaling up such easily accessible, and brief interventions. Several limitations were noted, including the lack of ecological momentary assessments to capture perceived stress more accurately, longer-term follow-up measures to evaluate sustainability of gains, and more diverse samples to evaluate transferability of the findings.

## Appendix

Multimedia Appendix 1 CONSORT-EHEALTH Checklist (V 1.6.1).

## Abbreviations

AES: App Engagement Scale
ANCOVA: analysis of covariance
CBT: cognitive behavioral therapy
CSE: coping self-efficacy
CSES: Coping Self-Efficacy Scale
GAD: Generalized Anxiety Disorder
mHealth: mobile health
PHQ: Patient Health Questionnaire
PM: psychological mindedness
PMS: Psychological Mindedness Scale
PSM: Psychological Stress Measure
RCT: randomized controlled trial
SGI: self-guided intervention

## Data Availability

Data supporting the findings of this study are available upon request from the corresponding author.
